# Psychosis in Parkinson’s disease: a clinical biomarker of disease stage and prognosis

**DOI:** 10.1192/j.eurpsy.2023.1985

**Published:** 2023-07-19

**Authors:** M. Pinho, D. O. Martins, P. S. Martins, L. Gomes, S. Carvalho

**Affiliations:** 1 Hospital de Magalhães Lemos, Porto, Portugal

## Abstract

**Introduction:**

Parkinson’s disease (PD) is a neurodegenerative disorder characterized by motor and nonmotor symptoms, the latter contributing significantly to morbidity, mortality, nursing home placement and quality of life.

**Objectives:**

We present a literature review about the impact of psychosis on PD’s prognosis.

**Methods:**

A literature review is performed on PUBMED, using the next keywords: "Parkinson’s disease”, “psychosis” and “prognosis”. We focused on data from systematic reviews, clinical trials and meta-analysis published in English on last 10 years.

**Results:**

Psychosis is a common feature of Parkinson’s disease, occurring in up to 30% of PD patients treated chronically with antiparkinsonian drugs. Visual hallucinations are the most common psychotic symptom observed, delusions being considerably less common and affecting only 5% of treated patients.

Positive symptoms in PD vary across its course: early in the disease, passage hallucinations, illusions and presence hallucinations occur; later, complete visual hallucinations, initially with good insight, then without insight.

Psychosis spectrum symptoms in early PD predict a decline in cognitive function at 2 years, especially visual hallucinations. There is an association between visual hallucinations and the subsequent emergence of dementia.

**Conclusions:**

Current evidence highlights the role of PD psychosis as a clinical biomarker of disease stage, distribution and future progression. Early recognition and treatment of psychotic symptoms improves disease’s outcomes.

**Disclosure of Interest:**

None Declared

